# Genetic determinants of lipid metabolism in cardioprotection: From mechanisms to clinical practice

**DOI:** 10.17305/bb.2025.13176

**Published:** 2025-12-04

**Authors:** Mia Manojlovic, Diana Mahmoud, Magdalena Pantic, Melaku Taye Amogne

**Affiliations:** 1 University of Novi Sad, Faculty of Medicine in Novi Sad, Novi Sad, Serbia; 2 Clinic for Endocrinology, Diabetes and Metabolic Disorders, University Clinical Center of Vojvodina, Novi Sad, Serbia; 3 Department of Endocrinology, Medical Faculty, Medical University Sofia, USHATE “Acad. Ivan Penchev”, Sofia, Bulgaria; 4 College of Health Sciences, Addis Ababa University, Department of Internal Medicine, Addis Ababa, Ethiopia

**Keywords:** Cardioprotective genetic determinants, lipids, atherosclerosis, hypocholesterolemia, mutation

## Abstract

Atherosclerotic cardiovascular diseases (ASCVD) continue to be the leading causes of morbidity and mortality globally. Disorders of lipoprotein metabolism contribute significantly to the development of atherosclerosis, which begins with the subendothelial retention of plasma-derived apolipoprotein B-containing lipoproteins, particularly low-density lipoprotein (LDL) and its remnants. Elevated LDL cholesterol levels and triglycerides, coupled with low high-density lipoprotein (HDL) cholesterol levels, are critical risk factors for ASCVD. Landmark epidemiological studies have identified dyslipidemia as a key modifiable risk factor for these diseases, elucidating the essential role of lipid abnormalities in atherogenesis and highlighting significant opportunities for cardiovascular disease prevention and risk stratification. Genetic epidemiology studies have shown that lifelong low levels of LDL due to genetic variations markedly reduce the risk of ASCVD. Recent advancements in lipid-lowering pharmacology are increasingly informed by genetic studies that reveal naturally occurring mutations offering lifelong cardioprotection. Furthermore, these genetic studies have facilitated the development of novel therapeutics and enhanced the prediction of potential side effects, variability in individual drug responses, and improved risk stratification. This narrative review article aims to summarize key genetic variants that influence lipid metabolism and examine their therapeutic potential in cardiovascular therapy. Given the central role of atherosclerosis in determining cardiovascular risk, it is vital to consider lipid metabolism in the context of genetic factors that affect individual susceptibility to hyperlipidemia. Defining cardioprotective genetic determinants is equally important, as it may provide a foundation for therapeutic strategies by emphasizing protective mechanisms.

## Introduction

Atherosclerotic cardiovascular diseases (ASCVD) remain the leading cause of morbidity and mortality globally, representing an increasing burden despite decades of therapeutic advancements. Recent estimates from the Global Burden of Disease (GBD) 2023 study indicate that the prevalence of ASCVD has more than doubled, rising from approximately 311 million cases in 1990 to 626 million in 2023. Concurrently, annual cardiovascular deaths increased from 13.1 million to 19.2 million, accounting for nearly one-third of all global mortality [1]. Furthermore, the 2024 Heart Disease and Stroke Statistics update from the American Heart Association (AHA) reports that 48.6% of U.S. adults aged 20 years and older are affected by ASCVD, which remains the leading cause of death in the United States, with a cardiovascular-related fatality occurring every 34 s [2].

Disorders of lipoprotein metabolism are a critical factor in the pathogenesis of atherosclerosis, which begins with the subendothelial retention of plasma-derived apolipoprotein B-containing lipoproteins, particularly low-density lipoprotein (LDL) and its remnants [3–5]. Elevated levels of LDL cholesterol (LDL-C) and triglycerides, along with decreased levels of high-density lipoprotein cholesterol (HDL-C), are significant risk factors for ASCVD [1]. Landmark studies in cardiovascular disease, including the Framingham Heart Study [6], the Seven Countries Study [7], the Multiple Risk Factor Intervention Trial (MRFIT) [8], the Atherosclerosis Risk in Communities (ARIC) Study [9], and the INTERHEART Study [10], have established dyslipidemia as a key modifiable risk factor for ASCVD. These studies elucidated the role of lipid abnormalities in atherosclerosis development and highlighted opportunities for cardiovascular disease prevention and risk assessment, thereby informing modern lipid management strategies. Subsequent genetic and Mendelian randomization studies have further reinforced the causal role of LDL-C in atherosclerotic disease, demonstrating that lifelong low levels of LDL-C, attributable to genetic variants, confer substantial protection against ASCVD [11–14].

Lipid-lowering therapies have been validated as effective interventions for reducing LDL-C and ASCVD risk. Robust evidence from the Cholesterol Treatment Trialists’ (CTT) Collaborators’ meta-analyses—comprehensive syntheses of data from multiple large-scale randomized controlled trials—has established statins as the cornerstone of lipid-lowering therapy, consistently demonstrating a proportional reduction in major vascular events per mmol/L reduction in LDL-C across diverse populations and risk groups [15–19]. When combined with statin therapy, ezetimibe achieves incremental LDL-C reduction and provides additional cardiovascular benefits, as evidenced by the IMProved Reduction of Outcomes: Vytorin Efficacy International Trial (IMPROVE-IT) [20]. Beyond statins and ezetimibe, proprotein convertase subtilisin/kexin type 9 (*PCSK9*) inhibitors, such as alirocumab and evolocumab, have shown significant LDL-C reductions and further cardiovascular risk mitigation in the FOURIER [21] and ODYSSEY OUTCOMES [22] trials. More recently, inclisiran—a small interfering RNA that inhibits *PCSK9* expression—has demonstrated sustained LDL-C lowering in patients with established ASCVD or those at equivalent risk, as shown in the ORION-10 and ORION-11 trials [23]. Bempedoic acid, offering statin-independent LDL-C lowering, has been shown in the CLEAR Outcomes trial to reduce major adverse cardiovascular events among statin-intolerant patients, highlighting its role as an alternative lipid-lowering strategy [24].

Advancements in dyslipidemia management are increasingly informed by genetic studies identifying naturally occurring mutations that confer lifelong cardiovascular protection through modulation of lipid metabolism. Such beneficial genetic variants often result in hypocholesterolemia. In contrast, observational associations between elevated HDL-C and lower cardiovascular risk have not been validated as causal in genetic analyses [25, 26]. Additionally, insights from human genetics have informed the development of novel therapeutics, improved predictions of interindividual variability in drug response, and enhanced cardiovascular risk stratification [27–30].

This review article aims to summarize key advantageous genetic variants influencing lipid metabolism and explore their therapeutic potential in cardiovascular therapy. Understanding these genetic insights is essential for advancing lipid management and further reducing cardiovascular risk.

## Methods

This article presents a narrative review of genetic variants in lipid metabolism that are associated with a favorable lipid profile and cardioprotection. This review format was selected due to the diverse types of evidence that require integrative interpretation. A comprehensive literature review was conducted using major biomedical databases (PubMed, Embase, Web of Science, and ClinicalTrials.gov). Search terms included gene names (e.g., “PCSK9,” “ANGPTL3,” “APOC3,” “CETP”), phenotype descriptors (e.g., “loss-of-function,” “gain-of-function,” “low cholesterol,” “low triglycerides,” “increased HDL”), and therapeutic terms (e.g., “PCSK9 inhibitor,” “APOC3 inhibitor,” “CETP inhibitor,” “CRISPR”). Our inclusion prioritization was as follows: cardiovascular outcomes trials > large prospective cohorts/Mendelian randomization > mechanistic/functional genomics > preclinical/animal studies. The final search was conducted on November 8, 2025. We employed a thematic, gene- and pathway-based narrative synthesis, organizing the evidence according to major phenotypes.

## Genetic insights into hypocholesterolemia

LDL-C levels are influenced by both genetic and environmental factors, with LDL-C being a derivative of very-LDL cholesterol (VLDL-C) that accounts for approximately 70% of plasma cholesterol [31]. While previous research has primarily focused on identifying mutations that elevate LDL-C levels, thereby increasing cardiovascular risk for carriers, contemporary scientific efforts should also explore naturally occurring mutations associated with hypocholesterolemia. This exploration is essential for advancing our understanding of normal lipid metabolism. Prior studies have identified five genes linked to low (<50 mg/dL) or extremely low (<20 mg/dL) LDL-C levels, primarily as monogenic causes [32]. These include familial hypobetalipoproteinemia (FHBL) due to mutations in apolipoprotein B (*APOB*), abetalipoproteinemia caused by mutations in microsomal triglyceride transfer protein (*MTTP*), hypocholesterolemia resulting from mutations in *PCSK9*, chylomicron retention disease due to mutations in *SAR1B*, and combined hypolipidemia caused by mutations in angiopoietin-like 3 (*ANGPTL3*) [32, 33]. The phenotypic presentation of these conditions varies widely, ranging from the cardioprotective effects of low LDL-C levels observed in individuals with single heterozygous mutations in *APOB*, *ANGPTL3*, and *PCSK9*, to the polymorphic manifestations of biallelic mutations, which typically present early in childhood and include failure to thrive, malabsorption, steatorrhea, sensory neuropathies, and retinal degeneration [32, 34].

### *APOB* mutations

A study published in 2021 by Jakubowski et al. [32], aimed to identify monogenic and polygenic causes of low and extremely low LDL-C levels through whole-exome sequencing (WES) and included a cohort of nine patients. The study found that truncating mutations in the *APOB* gene were the most prevalent cause (Table 1). In contrast, mutations in other genes, such as *ANGPTL3* and *PCSK9*, which had previously been classified as benign or of uncertain significance, were detected in a minority of patients. The cohort consisted of relatively healthy adult participants, excluding individuals with chylomicron retention disease and abetalipoproteinemia, resulting in a lack of uniform phenotypic presentation. Notably, three out of nine patients exhibited diarrhea and hepatic steatosis, attributed to reduced apoB secretion, while two patients had vitamin D deficiency.

**Table 1:** Genetic variants in low-cholesterol phenotypes

*APOB gene*
*Truncating mutations (Frameshift)*		
**Mutation**	**Type of mutation**	**Reference**
c.1250delT	Frameshift deletion	[31]
c.2157delG	Frameshift deletion	[32]
c.2855delT	Frameshift deletion	[32]
c.4186_4187delGT	Frameshift deletion	[31]
c.4800_4801insC	Frameshift insertion	[31]
c.7158delA	Frameshift deletion	[31]
c.8771delC	Frameshift deletion	[31]
c.10728dupC	Frameshift duplication	[31]
c.10848delT	Frameshift deletion	[32]
c.2817-75_3121+75del1064bp	Large deletion	[32]
*Truncating mutations (Nonsense)*		
c.409G>T	Nonsense	[32]
R2486X	Nonsense	[31]
Y1719X	Nonsense	[31]
*Splice-site mutations*		
537+1 G>T	Splice-site variant	[31]
*Missense mutations*		
R463W	Missense	[38]
L343V	Missense	[39]
P1116S	Missense	[31]
P2794L	Missense	[31]
*In-frame variants*		
c.6636_6638delTGA	In-frame deletion	[31]
*PCKS9 gene*		
**Mutation**	**Type of mutation**	**Reference**
Y142X	LOF	[43]
C679X	LOF	[43]
R46L	LOF	[45]
E144K	LOF	[40]
C378W	LOF	[40]
G106R	LOF	[42]
R237W	LOF	[42]
*LDLR mutations*		
**Mutation**	**Type of mutation**	**Reference**
del2.5	GOF	[47]

Abbreviations: APOB: Apolipoprotein B; PCSK9: Proprotein convertase subtilisin/kexin type 9; LDLR: Low-density lipoprotein receptor; LOF: Loss-of-function; GOF: Gain-of-function.

Prior to inclusion, individuals with potential secondary causes of hypocholesterolemia—such as adrenal insufficiency, hyperthyroidism, severe liver disease, chronic infections, strict vegetarian or vegan diets, intestinal fat malabsorption, malnutrition, and active or prior malignancy—were excluded, as were those receiving lipid-lowering therapy. This rigorous selection process differentiates the study from previous population-based investigations, particularly concerning very low levels of LDL-C. However, a noted limitation of the study is that LDL-C levels were calculated using the Friedewald formula rather than directly measured through ultracentrifugation. The authors emphasized the importance of clinical monitoring for patients with *APOB* truncating mutations, particularly regarding hepatic steatosis, due to its potential progression to cirrhosis and hepatocellular carcinoma (HCC). Existing literature indicates that LDL-C levels in heterozygous individuals with *APOB* truncating mutations are approximately 25%–30% of normal values [35].

Furthermore, an earlier study by Leren and Berge [31] demonstrated that frameshift mutations in the *APOB* gene are more prevalent than nonsense mutations as causes of truncated *APOB*. Proposed mechanisms include reduced LDL production due to nonsense-mediated decay and enhanced LDL catabolism, as truncated apoB proteins shorter than apoB-70 are rapidly cleared by proximal renal cells via the megalin receptor [36]. Additionally, upregulation of the LDL receptor (LDLR) in response to low cholesterol levels may contribute to the accelerated clearance of full-length apoB-100 produced by the normal allele [37]. Among the 93 unrelated subjects in this investigation, one individual carried a splice-site mutation (537+1 G>T) in the *APOB* gene. Several frameshift mutations were also identified, including 1250delT in exon 10, a two-base-pair deletion (4186-4187delGT) in exon 25, a one-base-pair insertion (4800-4801insC) in exon 26, 7158delA in exon 26, a one-base-pair deletion (8771delC) in exon 26, and a one-base-pair duplication (10728dupC) in exon 26. Regarding in-frame variants, a three-base-pair deletion (6636-6638delTGA) in exon 26 was detected. Additionally, some individuals harbored nonsense mutations, including Y1719X and R2486X [31]. Missense mutations in the *APOB* gene are considered a rare cause of autosomal dominant hypocholesterolemia; however, several variants have been proposed to possess pathogenic potential, including R463W [38], L343V [39], P1116S, and P2794L [31].

### *PCSK9* mutations

In the context of *PCSK9*, approximately 300 loss-of-function (LOF) mutations have been identified as contributors to low cholesterol levels across diverse human populations [40–42]. Among the initial *PCSK9* LOF mutations discovered in African American individuals were Y142X and C679X [43], followed by R237W and R46L, which were reported in South African, New Zealand, and European White populations [44, 45]. Additionally, E144K and C378W mutations have been identified among Chinese Uyghurs [40]. Berge et al. [42] documented the presence of R46L, G106R, and R237W mutations in a cohort of 38 unrelated individuals with hypocholesterolemia, all exhibiting cholesterol levels below 154.68 mg/dL. Their findings indicated a statistically significant higher frequency of these mutations compared to 441 hypercholesterolemic patients, suggesting that these variants are not merely benign polymorphisms.

In a second cohort consisting of nine familial hypercholesterolemia (FH) patients who responded favorably to statin therapy, one patient was heterozygous for R46L and another for N157K [42]. Additionally, the extensive Dallas Heart Study, which screened 3553 participants, identified Y142X, C679X, and R46L among individuals in the lowest 25th percentile for cholesterol levels [43]. Moreover, the study by Leren and Berge [31] revealed that six out of 93 participants were heterozygous for R46L (four subjects), G106R (one subject), and R237W (one subject).

### *LDLR* mutations

Regarding *LDLR*, gain-of-function (GOF) mutations are believed to contribute to reduced LDL-C levels and provide cardiovascular protection. Existing literature describes sequence variants in *LDLR* that result in modest GOF mutations with a pronounced effect on LDL-C levels [46, 47]. For instance, a 2.5 kilobase (kb) deletion (del2.5) in the 3′ untranslated region (UTR) of *LDLR* was detected in a study utilizing whole-genome sequencing data from 43,202 Icelandic individuals. This deletion was identified as a cause of a substantial 74% reduction in circulating LDL-C levels, attributed to enhanced expression of LDLR. Furthermore, the authors hypothesized that the elevated intracellular cholesterol levels resulting from increased *LDLR* activity might downregulate the sterol regulatory element-binding protein (SREBP) pathway. This hypothesis is supported by their observation of modestly lower concentrations of circulating *PCSK9* in del2.5 carriers. The authors also emphasized that this mutation enhances the clearance of LDL from circulation without affecting lipoprotein synthesis or secretion, thereby likely reducing the risk of hepatic steatosis in these individuals [47].

## Genetic variants associated with low triglyceride levels

Severe hypertriglyceridemia is increasingly recognized as a genetically influenced disorder of triglyceride-rich lipoprotein (TRL) metabolism, resulting from both rare monogenic defects and more prevalent polygenic factors that enhance triglyceride production and/or impair clearance [48]. The regulatory pathways responsible for pathological triglyceride elevation—such as lipoprotein lipase (LPL) activity and its modulators—are also the sites where naturally occurring protective variants exert their effects in the opposing direction. *LPL*, a lipase that hydrolyzes triglycerides in very-LDLs (VLDL) and chylomicrons, serves as a rate-limiting factor for triglyceride clearance. LPL activity is tightly regulated at the post-translational level by apolipoproteins and angiopoietin-like proteins (ANGPTLs) [49]. Notably, apolipoprotein C3 (APOC3) and ANGPTL proteins inhibit *LPL* activity, thereby elevating circulating triglyceride levels [50].

The translational implications of these findings are significant, as plasma triglyceride levels contribute to cardiovascular risk independently of LDL-C concentrations [51]. However, the 2025 Focused Update of the 2019 ESC/EAS Guidelines for the management of dyslipidemias continues to endorse statins as the primary pharmacological intervention for reducing cardiovascular risk in high-risk populations. Conversely, despite ongoing research, evidence supporting the efficacy of triglyceride-lowering therapies, such as fibrates, in decreasing ASCVD events remains inconclusive [52, 53]. Given the persistence of this narrative over recent years, it is instructive to revisit studies utilizing genetic analyses to assess the impact of variants associated with lower triglyceride levels on cardiovascular outcomes. For instance, Ference et al. [54] employed Mendelian randomization to compare the associations of triglyceride-lowering *LPL* variants and LDL-C-lowering *LDLR* variants with cardiovascular disease risk per unit reduction in apoB-containing lipoproteins. Their analysis aimed to elucidate the potential comparative clinical benefits of targeting plasma triglycerides relative to LDL-C. The results demonstrated that both triglyceride-lowering *LPL* variants and LDL-C-lowering *LDLR* variants were associated with similar reductions in coronary artery disease (CAD) risk per unit decrease in apoB-containing particles. Furthermore, multiple variants within genes encoding targets of both triglyceride-lowering therapies (via the LPL pathway) and LDL-C-lowering therapies (via the LDLR pathway) exhibited comparable associations with lower CAD risk per unit reduction in plasma apoB. Genetic scores comprising additional variants associated with triglycerides, LDL-C, or both also indicated similar reductions in CAD risk per unit decrease in apoB, even when the direction of change in triglycerides and LDL-C varied [54]. Collectively, these results suggest that all apoB-containing lipoproteins, including triglyceride-rich VLDL particles and their metabolic remnants, as well as LDL particles, contribute approximately equally to cardiovascular risk on a per-particle basis. The data imply that the risk posed by apoB-containing particles is primarily determined by their circulating concentration, which influences the number of particles retained in the arterial wall, irrespective of whether they predominantly transport cholesterol or triglycerides. Consequently, future randomized trials investigating novel triglyceride-lowering therapies should consider the overall reduction in apoB-containing particles to evaluate cardiovascular benefits [54, 55].

Genetic epidemiology studies have identified naturally occurring GOF variants in LPL and LOF variants in its intravascular inhibitors (*APOC3, ANGPTL3*, and *ANGPTL4*) that are associated with lower triglyceride levels and reduced coronary disease risk [56–58]. These findings emphasize the central role of *LPL* regulatory pathways in triglyceride metabolism and provide a mechanistic foundation for developing novel therapeutic strategies targeting hypertriglyceridemia. Additionally, genetic studies support a causal relationship between elevated concentrations of TRLs and low-grade inflammation [59].

### *LPL* mutations

A GOF mutation in the *LPL* gene, such as the S447X variant, is associated with enhanced lipolytic function, resulting in an anti-atherogenic lipid profile characterized by lower plasma triglyceride levels and potentially reduced risk of CAD [60]. A Human Genome Epidemiology (HuGE) association review and meta-analysis, which analyzed data from over 70,000 participants across multiple studies, confirmed a connection between *LPL* GOF variants (HindIII [rs320], S447X [rs328]) and modestly advantageous lipid profiles linked to lower CVD risk [61].

Additionally, a large-scale exome-wide association analysis assessed 54,003 coding sequence variants across 13,715 human genes in up to 72,868 individuals with CAD and 120,770 controls. This analysis revealed several low-frequency coding variants associated with either increased susceptibility to or protection from CAD, identified through DNA genotyping. Notably, a rare missense variant in the *LPL* gene (p.D36N, also known as p.D9N) was linked to an elevated CAD risk, while a nonsense variant in *LPL* (p.S447) was associated with a significant reduction in CAD risk. The researchers emphasized that, contrary to the typical outcome where a premature stop codon results in LOF, this particular nonsense variant—located in the penultimate codon of the *LPL* gene—paradoxically enhances enzyme activity, representing a GOF mutation. These findings underscore the critical role of the LPL pathway in modulating susceptibility to CAD [62].

### *APOC3* mutations

A study by the TG and HDL Working Group of the Exome Sequencing Project aimed to sequence the protein-coding regions of 18,666 genes in 3734 individuals and evaluate rare mutations in the coding sequence potentially associated with plasma triglyceride levels. For mutations linked to triglyceride levels, the researchers later examined their relationship with CHD risk in a cohort of 110,970 individuals. They found that nearly 1 in 150 individuals were heterozygous carriers of at least one of four rare mutations in the gene encoding *APOC3*. The aggregate of these mutations was associated with reduced circulating triglyceride concentrations, including two splice-site mutations (IVS2+1G→A and IVS3+1G→T) and a nonsense mutation (R19X) identified as LOF mutations, along with one missense mutation (A43T). Carriers exhibited *APOC3* circulating levels that were 46% lower than noncarriers and had triglyceride levels that were 39% lower. Overall, the risk of CHD was 40% lower among carriers compared to noncarriers [56].

In a population-based cohort of 75,725 Danish individuals, heterozygous carriers of *APOC3* LOF mutations demonstrated a 44% reduction in plasma triglyceride levels compared to noncarriers. Furthermore, these carriers had a 41% lower risk of ischemic heart disease and a 36% lower risk of ischemic vascular disease overall [63].

These findings suggest that *APOC3* LOF mutations confer significant cardioprotective effects, likely due to their role in reducing triglyceride levels.

### *ANGPTL* mutations

As previously mentioned, a large-scale exome-wide association analysis evaluated 54,003 coding sequence variants, identifying 10 variants predicted to cause LOF mutations in the *ANGPTL4* gene. The study found that carriers of these LOF mutations had triglyceride levels 35% lower than those of noncarriers, while differences in LDL and HDL cholesterol levels remained insignificant. Additionally, carriers of these mutations exhibited a 53% decreased risk of CAD. The analysis provided strong evidence supporting a link between the p.E40K variant and reduced CAD risk, a hypothesis previously suggested in smaller studies, along with *in vitro* and *in vivo* experiments indicating potential destabilization of ANGPTL4 after secretion, leading to increased LPL activity [62].

Another extensive genetic association study that combined data from large population-based cohorts, including both case-control and prospective studies, found that inactivating mutations in *ANGPTL4* were associated with 13% lower triglyceride levels and a 19% lower risk of CAD compared to noncarriers [57]. Besides the canonical *ANGPTL4* LOF variant E40K, the T266M missense variant was associated with a smaller triglyceride-lowering effect [64, 65].

A large-scale genetic dataset from the DiscovEHR study—a comprehensive genetic association and pharmacologic intervention study integrating human genetic epidemiology, population-based cohort analysis, and preclinical animal models—revealed that individuals carrying LOF variants in the *ANGPTL3* gene exhibited significantly lower plasma triglyceride levels and a reduced risk of CAD. Specifically, the study examined the relationship between individual and aggregated LOF variants in *ANGPTL3* and median serum lipid levels among over 45,000 participants with documented lipid measurements. Individuals carrying these LOF variants exhibited substantially lower triglyceride concentrations (27%) and modest reductions in LDL (9%) and HDL (4%) cholesterol levels compared to noncarriers, even after adjusting for relevant covariates. The most commonly verified *ANGPTL3* LOF variants included p.Ser17Ter, p.Asn121fs (found in 91 individuals), and a novel rare frameshift insertion-deletion variant (p.Asn147fs). A splice region variant, c.495+6T→C, was also identified. Among the study cohort, 246 individuals were found to be heterozygous carriers of LOF variants, corresponding to an estimated frequency of one carrier for every 237 participants of European ancestry in the DiscovEHR dataset. Consistent with the low frequency of these alleles, no homozygous or compound heterozygous individuals were observed [66]. Individuals homozygous for inactivating *ANGPTL3* variants exhibited profound combined hypolipidemia, with up to 71% lower plasma triglycerides and 67% lower LDL-C compared to noncarriers [67].

Regarding naturally occurring *ANGPTL8* variants, a protein-truncating variant (rs145464906; c.361C>T; p.Q121X) was identified that was associated with lower triglyceride and higher HDL-C levels in a large exome-array-based genetic association study of 56,538 individuals from multiple population-based and case-control cohorts [68].

Table 2 summarizes the key features of genetic variants associated with low triglyceride levels.

**Table 2:** Key features of genetic variants associated with low triglyceride levels

**Mutations**	**Key variants**	**Mechanism**	**Translational potential**
***APOC3*** LOF mutations	R19X A43T IVS2+1G →A IVS3+1G →T	Increased *LPL* activity → enhanced triglyceride clearance → reduced atherogenic remnant cholesterol particles	Development of *APOC3* inhibitors volanesorsen [96], olezarsen [121] and plozasiran [102]
***ANGPTL*** LOF mutations	***ANGPTL3*** p.Ser17Ter p.Asn121fs p.Asn147fs c.495+6T→C ***ANGPTL8*** p.Q121X ***ANGPTL4*** E40K T266M	Disinhibition of *LPL* and subsequent hydrolysis of triglyceride-rich lipoproteins	ANGPTL3 targeting agents: Evinacumab [103] and Zodasiran [107]. Vupanorsen-discontinued [106]
***LPL*** GOF mutations	S447X	Increased *LPL* activity enhances triglyceride clearance and reduces CVD risk	A proof-of-concept data from alipogene tiparvovec in *LPL* deficiency [122]

Abbreviations: LPL: Lipoprotein lipase; CVD: Cardiovascular disease; APOC3: Apolipoprotein C3; ANGPTL: Angiopoietin-like protein; LOF: Loss-of-function; GOF: Gain-of-function.

## Genetic variations that lead to increased HDL-C levels

Epidemiological studies have established an inverse association between HDL-C levels and the risk of ASCVD [69, 70]. A meta-analysis of extensive prospective cardiovascular cohort studies indicates a consistent inverse relationship, independent of other cardiovascular risk factors, where each 1 mg/dL increase in HDL-C reduces the risk of CAD by 2%–3% [71]. The atheroprotective effects of HDL-C are believed to be mediated through reverse cholesterol transport (RCT), anti-inflammatory properties, antioxidant effects, and endothelial protection [72–74].

Despite the beneficial effects observed in observational studies, the “HDL hypothesis” has not been conclusively validated in numerous genetic and pharmacological studies [75]. A 2009 meta-analysis encompassing 108 randomized trials with 299,310 participants investigating HDL-raising therapies found no significant association between treatment-induced changes in HDL-C and CAD mortality, CAD events, or all-cause mortality, even after adjusting for changes in LDL-C [76].

Although the initially promising HDL hypothesis did not reliably translate into cardioprotective effects, genetic variations impacting HDL metabolism offer crucial insights into cardiovascular risk modulation. Among the most studied genes are cholesteryl ester transfer protein (*CETP*) and hepatic lipase (*LIPC*), whose mutations result in elevated HDL-C levels.

### *CETP* deficiency mutations

Certain genetic variants in the *CETP* gene are associated with reduced CETP activity, which leads to higher HDL-C concentrations. A 2008 systematic review identified three *CETP* gene polymorphisms (TaqIB, I405V, and --629C>A) commonly associated with inhibited CETP activity and modestly elevated HDL-C levels [77]. Despite the increase in HDL-C concentration, these *CETP* polymorphisms did not correlate with reduced CVD risk.

A study involving a substantial subset of participants from the Framingham Offspring Study, who were genotyped for the *CETP* TaqIB polymorphism, demonstrated that the B2 allele is linked to lower *CETP* activity and higher HDL-C levels. However, this lipoprotein profile was not significantly associated with reduced CHD risk, particularly among women [78].

A Mendelian randomization analysis of the *CETP* gene from the Copenhagen City Heart Study suggests that genetic variants associated with reduced *CETP* activity correlate with increased HDL-C, decreased LDL-C, and a lower risk of atherosclerotic cardiovascular events [79].

Moreover, a cross-sectional study utilizing data from the Honolulu Heart Program, which included a cohort of 3469 Japanese–American men, revealed that two *CETP* gene mutations (D442G and intron 14G:A) were associated with higher HDL-C levels. A higher prevalence of CHD was observed among mutation carriers, particularly those with HDL-C levels between 41 and 60 mg/dL [80].

The translational potential of *CETP* inhibition remains limited, as trials involving *CETP* inhibitors such as anacetrapib [81], dalcetrapib [82], and torcetrapib [83] have failed due to lack of benefit or adverse effects. In the REVEAL trial, anacetrapib demonstrated positive cardiovascular outcomes when added to intensive statin therapy, with no safety concerns [84]. Despite achieving its primary endpoint, the absolute benefit was deemed modest, leading the sponsor not to pursue regulatory approval for anacetrapib.

### Other mutations that cause increased HDL-C

LOF mutations in hepatic lipase lead to increased HDL-C levels by impairing the catabolism of large HDL particles [85]. Genetic studies have indicated inconsistent associations with CAD risk [86].

LOF mutations in endothelial lipase (*EL*), a member of the lipase gene family, may also be associated with elevated HDL-C levels [87].

Scavenger receptor class B type I (*SR-BI*) is another candidate gene in which LOF mutations result in significantly increased HDL-C levels and a markedly decreased risk of CHD [88].

In addition to very low plasma triglyceride levels, *ApoC-III* LOF mutations are associated with elevated HDL-C and a reduction in the risk of CHD compared to non-carriers [56, 89].

**Figure 1.**
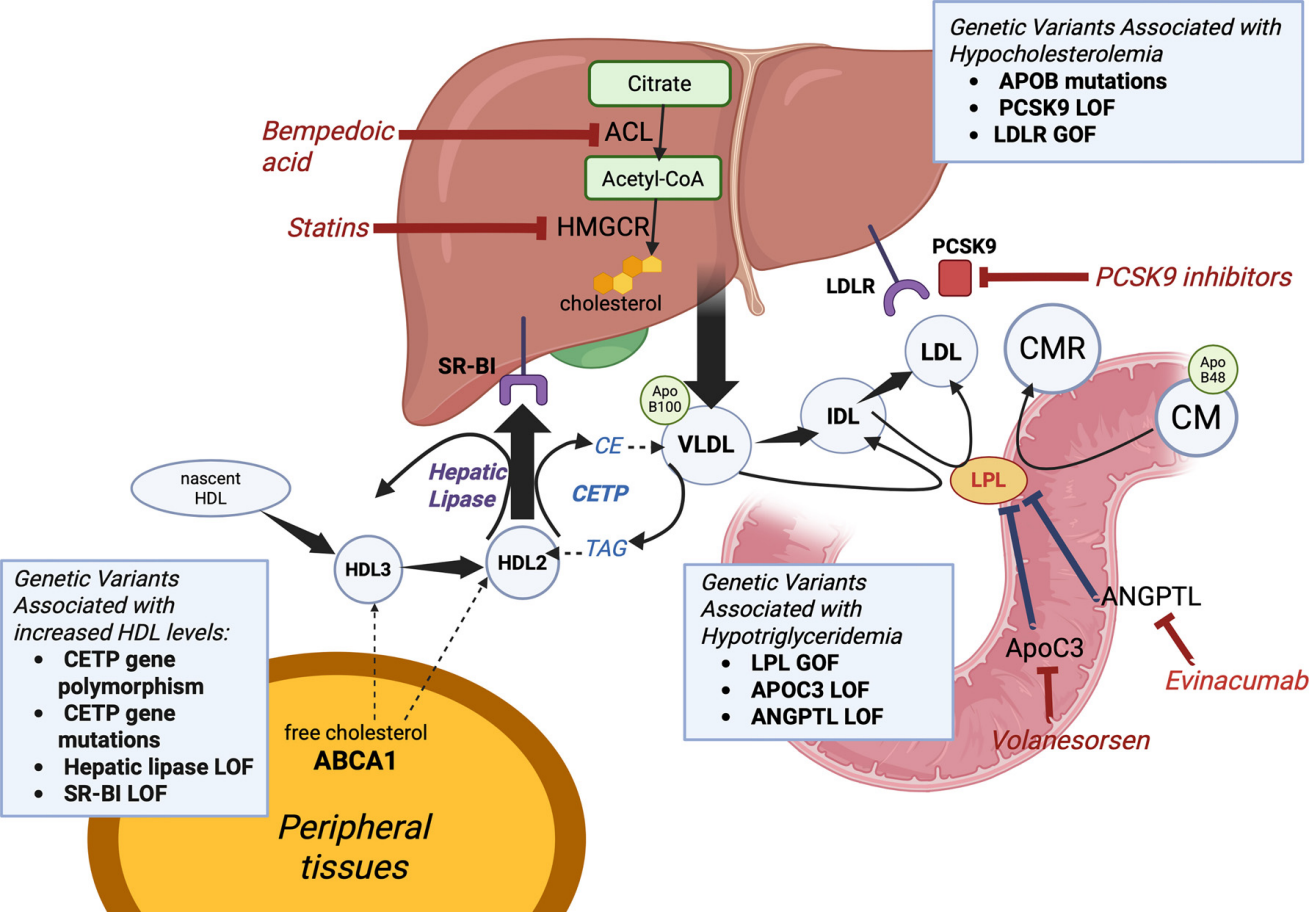
**Genetic determinants of lipid metabolism and targets for lipid-lowering therapies**. In the liver, cholesterol is synthesized from citrate through ACLY and HMGCR, which are targeted by bempedoic acid and statins, respectively. Cholesterol is incorporated into VLDL, which contain Apo B100, and are secreted into circulation. VLDL is progressively metabolized into IDL and LDL. LDL is cleared from circulation via *LDLR* on hepatocytes. *PCSK9* binds to *LDLR*s, leading to their degradation. *PCSK9* inhibitors enhance *LDLR* recycling, increasing LDL clearance. Intestinal mucosal cells secrete the TAG-rich chylomicrons, produced primarily from dietary lipids. LPL degrades the TAG in the chylomicrons, subsequently CMRs-cholesteryl ester rich chylomicrons, are formed. LPL activity is inhibited by *ANGPTL* and *ApoC3*. They are targeted by monoclonal antibodies evinacumab (anti-*ANGPTL3*), and antisense oligonucleotides volanesorsen (targeting *ApoC3*), enhancing triglyceride clearance. HDL particles are involved in the reverse cholesterol transport. Free cholesterol from peripheral tissues is exported via ABCA1 to nascent HDL, which goes on to mature through remodeling into HDL2 and HDL3. *CETP* mediates an exchange of lipids by transferring CE from HDL to VLDL/LDL; and TAG from VLDL/LDL to HDL. Hepatic lipase hydrolyses triglycerides and phospholipids in HDL2, transforming it back to HDL3 and making it ready to repeat the cholesterol collection process. Mature HDL delivers cholesterol to the liver through *SR-BI*, completing the reverse transport pathway. An indirect pathway for delivery of cholesterol to the liver also occurs, where the cholesteryl esters are transferred from HDL to VLDL/LDL by *CETP* and eventually delivered to the liver where these particles are taken up by hepatic *LDLR*. Together, these mechanisms regulate lipid metabolism and serve as targets for various lipid-lowering therapies aimed at reducing cardiovascular risk. Abbreviations: ACLY: Adenosine triphosphate citrate lyase; HMGCR: HMG-CoA reductase; VLDL: Very low-density lipoprotein; Apo: Apolipoprotein; IDL: Intermediate-density lipoprotein; LDL: Low-density lipoprotein; LDLR: Low-density lipoprotein receptor; PCSK9: Proprotein convertase subtilisin/kexin type 9; TAG: Triacylgyceride; CM: Chylomicron; LPL: Lipoprotein lipase; CMR: Chylomicron remnant; ANGPTL: Angiopoietin-like proteins; HDL: High-density lipoprotein; ABCA1: ATP-binding cassette transporter A1; CETP: Cholesteryl ester transfer protein; CE: Cholesteryl esters; SR-BI: Scavenger receptor class B type I; GOF: Gain-of-function mutations; LOF: Loss-of-function mutations. Created in BioRender. Mahmoud, D. (2025) https://BioRender.com/6soka2d.

Some genetic determinants of lipid metabolism, as well as key targets of lipid-lowering therapies, are illustrated in Figure 1.

## Potential therapeutic implications and future directions

Cardioprotective genetic variants of lipid-regulatory genes provide valuable insights into lipid pathways and cardiovascular disease risk. The association of these genetic lipid phenotypes with a reduced lifetime risk of ASCVD has laid the groundwork for the development of various lipid-lowering therapies. Variants that truncate or reduce apoB production, LOF mutations in *PCSK9*, and increased activity of the LDLR pathway all contribute to lower LDL-C levels from birth and are associated with significantly reduced atherosclerotic cardiovascular events throughout life [32, 34].

Among hypocholesterolemic genetic variants, studies on *PCSK9* have notably transformed lipid management into targeted therapeutics. Carriers of *PCSK9* LOF variants exhibit significantly lower LDL-C levels and a markedly reduced incidence of cardiovascular disease [90, 91]. These findings regarding the role of PCSK9 in lipid metabolism have led to the development of monoclonal antibodies targeting PCSK9 [92, 93] and, more recently, RNA-based therapies such as inclisiran, which silences hepatic *PCSK9* expression [94, 95]. PCSK9 inhibitors have expanded lipid-lowering therapeutic options beyond statins and ezetimibe, offering less frequent dosing and a favorable safety profile.

Similarly, genetic variants associated with low triglyceride levels have informed the development of treatments aimed at managing triglyceride levels. *APOC3*-targeted therapies represent a rational approach to treating severe hypertriglyceridemia and reducing triglyceride-rich remnants in patients with a high ASCVD burden. Volanesorsen, an antisense oligonucleotide (ASO), significantly reduced triglyceride levels and pancreatitis events in three randomized clinical trials: the Phase 3 APPROACH trial [96], the Phase 3 COMPASS trial [97], and the Phase 2/3 BROADEN study [98]. In light of these findings, the 2025 ESC/EAS focused update on dyslipidemia management recommends considering volanesorsen for patients with severe hypertriglyceridemia due to genetically confirmed familial chylomicronemia syndrome (FCS) [52]. Olezarsen, another ASO targeting *APOC3*, demonstrated significant reductions in triglycerides and a lower incidence of acute pancreatitis in the Phase 3 BALANCE trial [99] and has received regulatory approval in both the United States and the European Union for the treatment of FCS [100, 101]. Plozasiran, an siRNA targeting *APOC3*, has also shown substantial reductions in triglyceride levels and a decreased incidence of pancreatitis in the Phase 3 PALISADE trial [102] and is currently under regulatory review, potentially expanding future therapeutic options for FCS.

Therapies targeting ANGPTL3 have also been shown to effectively reduce triglyceride levels. Monoclonal antibody inhibition of ANGPTL3 with evinacumab has demonstrated reductions in triglycerides and TRLs across multiple studies, including the Phase 3 ELIPSE HoFH trial [103], a Phase 2 trial in refractory hypercholesterolemia (NCT03175367) [104], and a Phase 2 trial in severe hypertriglyceridemia (NCT03452228) [105]. Evinacumab has been approved for clinical use in patients with homozygous FH (HoFH) since 2021. Vupanorsen, an ASO targeting *ANGPTL3*, significantly reduced triglycerides and LDL-C in the Phase 2b TRANSLATE-TIMI 70 trial; however, its clinical development was discontinued in 2022 due to concerns regarding hepatic safety [106]. Zodasiran, an siRNA targeting *ANGPTL3*, has also shown significant reductions in triglycerides and LDL-C in the Phase 2b ARCHES-2 trial in adults with mixed hyperlipidemia [107], with further development ongoing, including the Phase 3 YOSEMITE trial evaluating the efficacy and safety of zodasiran in adolescents and adults with HoFH.

The next frontier in treating dyslipidemia extends beyond conventional pharmacotherapy to encompass the emerging field of gene-editing therapies. Recent years have revealed the potential of gene editing to reduce LDL-C levels, particularly through preclinical studies. This innovative tool permanently alters genetic foundations, decreasing disease susceptibility. However, confirmation of safety is still awaited before integration into clinical practice.

Gene GOF and LOF modifications are promising targets for clustered regularly interspaced short palindromic repeats (CRISPR)/CRISPR-associated protein 9 (Cas9) gene editing strategies, which can induce changes at specific nucleotide positions within the target DNA sequence. For optimal therapeutic efficacy, both Cas9 protein and CRISPR guide RNA must be present in the target cells, such as hepatocytes [108]. Consequently, there has been a shift from using adeno-associated virus (AAV) vectors, limited by cargo capacity, to lipid nanoparticles, which serve as biodegradable carriers with enhanced transport capacity and an improved safety profile. Lipid nanoparticles can be enriched with N-acetylgalactosamine (GalNAc), a high-affinity ligand for the asialoglycoprotein receptor on hepatocytes, ensuring targeted delivery without dependence on LDLR functionality [108, 109].

The established inverse relationship between lower LDL-C levels and reduced coronary heart disease (CHD) risk is reinforced by the discovery of *PCSK9* LOF variants, which provide compelling genetic evidence for this association. Specifically, nonsense mutations reducing plasma LDL-C by approximately 40 mg/dL (1.0 mmol/L) correlate with an 88% reduction in CHD incidence. In contrast, carriers of the *PCSK9* R46L allele, which lowers LDL-C by about 20 mg/dL (0.5 mmol/L), exhibit a 50% reduction in risk. These findings have significantly motivated research aimed at targeting *PCSK9* for the development of novel lipid-lowering and gene-based therapies [14]. A study by Ran et al. [110] demonstrated that using AAV as a vector along with smaller Cas9 protein variants from *Staphylococcus aureus* resulted in genetically modified PCSK9 levels reduced by up to 95%, leading to a total cholesterol decrease of approximately 40%. This research followed pilot studies by Ding et al. [111] and subsequent investigations by Chadwick et al. [112], both focusing on *PCSK9* gene targeting. Jiang et al. [113] explored lipid nanoparticles to mitigate immunogenicity and oncogenicity in gene therapy, finding that intravenous administration of Cas9 mRNA and single-guide RNA in lipid nanoparticles to C57BL/6 mice resulted in a significant reduction in LDL-C levels without liver damage. Subsequently, Zhang et al. [114] used a lipid shell anionic complex coated with 4-aminophenyl β-d-galactopyranoside-modified polyethylene glycol phospholipids, comprising two gene-targeting components (Gal-LGCP nanoparticles), confirming their effectiveness in lowering LDL-C levels. Despite the known mutation in the *LDLR* gene as the cause of FH, few studies have addressed CRISPR-based gene editing aimed at correcting this genetic defect. One notable study demonstrated the reversibility of the functional defect in the *LDLR* caused by the E208X LOF mutation in mice [115].

To enhance the clinical applicability of gene editing in lipidology, particularly for *PCSK9*, Wang et al. [116] were among the first to conduct translational studies in non-human primates. They targeted the *PCSK9* gene using the meganucleases M1 and M2, delivered via an AAV serotype 8 vector (AAV8-M1PCSK9). This approach resulted in an 84% reduction in *PCSK9* levels and a 60% decrease in LDL-C sustained for over 200 days. However, despite its groundbreaking nature, the study revealed significant unintended genetic modifications associated with the M1 variant, clinically manifesting as chronic elevation of liver transaminases. To address these adverse effects, Rothgangl et al. [117] utilized lipid nanoparticles to deliver *PCSK9*-targeting single-guide RNA alongside 1-methoxyuridine-modified mRNA encoding an adenine base editor. This method achieved only modest reductions in circulating PCSK9 protein and LDL-C levels, specifically 39% and 19%, respectively, attributed in part to an immune response against the Cas9 protein. To improve efficiency, Musunuru et al. [118] employed an advanced generation of base editors, achieving a remarkable 90% reduction in PCSK9 levels and a subsequent 60% decrease in LDL-C. Notably, this was associated with only a transient elevation in liver enzymes lasting less than two weeks, suggesting that this experimental strategy does not provoke pathological immune cell activation in monkeys.

Given these findings, the pursuit of gene editing therapy for human populations is evident, as reflected in the initiation of clinical trials. For instance, clinical trial NCT05398029 involves VERVE Therapeutics evaluating its investigational therapy, VERVE-101, in patients with FH. Concurrently, CRISPR Therapeutics has optimized its preclinical candidate, CTX330, a PCSK9-targeting gene editing therapy, for future clinical application [108].

Moreover, the transition from preclinical research to clinical implementation has expanded beyond *PCSK9* to other hepatocyte-expressed genes, including *ANGPTL3*, with both VERVE Therapeutics and CRISPR Therapeutics exploring their therapeutic potential [119]. Recently, a phase 1, open-label trial evaluated CTX310, a CRISPR–Cas9 therapeutic featuring lipid-nanoparticle–encapsulated Cas9 mRNA and guide RNA designed for LOF edits in hepatic *ANGPTL3*. This therapy was administered to 15 individuals with refractory hypercholesterolemia, hypertriglyceridemia, or mixed dyslipidemia, providing the first clinical proof-of-concept for *in vivo* gene editing targeting this pathway, demonstrating reductions in circulating *ANGPTL3*. Nonetheless, caution is warranted in interpreting these findings due to the small sample size and short follow-up period, highlighting the need for larger, long-term trials to establish efficacy and safety fully [120].

It is important to acknowledge several limitations of this review. It is narrative in nature and did not apply a standardized risk-of-bias tool to all included studies. While efforts were made to include the most relevant and recent evidence, the rapidly evolving field of lipid genetics may mean that some newly published studies are not included. Given the inherent heterogeneity of the evidence base, we conducted a pathway-based synthesis rather than a quantitative synthesis or meta-analytic evaluation. Additionally, rarer variants, less-explored pathways, and negative or unpublished findings may be underrepresented. These limitations should be considered when interpreting the mechanistic insights and clinical implications presented in this review.

## Conclusion

Given the pivotal role of atherosclerosis in cardiovascular risk, it is essential to consider lipid metabolism within the context of genetic determinants influencing individual susceptibility to hyperlipidemias. Recognizing a broad spectrum of cardioprotective genetic determinants is equally crucial, as it may serve as a cornerstone for therapeutic implications by shifting the focus toward protective mechanisms. The therapeutic implications of cardioprotective lipid genetics are no longer speculative. In the coming years, the growing understanding of gene-editing therapy’s potential and feasibility appears increasingly confident, opening new avenues while simultaneously raising critical questions regarding long-term safety, cost-benefit analyses, and ethical considerations.
